# Efficacy of Sequential Therapy as the First-Line Treatment in the Eradication of Helicobacter pylori

**DOI:** 10.7759/cureus.45593

**Published:** 2023-09-20

**Authors:** Yonas Tamene, Shefali P Mody, Kaiser O Sadiq, Yogamba M Shivakumar, Eshwar Burra, Kamran Shahid, Tuheen Sankar Nath

**Affiliations:** 1 Internal Medicine, California Institute of Behavioral Neurosciences & Psychology, Fairfield, USA; 2 Medicine, California Institute of Behavioral Neurosciences & Psychology, Fairfield, USA; 3 Surgery, Plexus Neuro and Stem Cell Research Centre, Bangalore, IND; 4 Internal Medicine, Government Medical College, Nizamabad, IND; 5 Internal Medicine/Family Medicine, California Institute of Behavioral Neurosciences & Psychology, Fairfield, USA; 6 Clinical Research, California Institute of Behavioral Neurosciences & Psychology, Fairfield, USA

**Keywords:** h. pylori eradication, h. pylori, h. pylori infection, helicobacter pylori, h. pylori antibotic

## Abstract

The Helicobacter pylori infection is a significant issue in global health as it is associated with a range of gastrointestinal disorders and an elevated likelihood of developing stomach cancer. The declining efficacy of standard triple therapy (TT) as the recommended treatment can be attributed to the emergence of drug-resistant strains. Sequential therapy (ST) has been recognized as an alternative approach, wherein a combination of proton-pump inhibitor (PPI) and amoxicillin is administered for the initial five days, followed by a combination of PPI, clarithromycin, and metronidazole for the subsequent five days. In this comprehensive systematic review and meta-analysis, we have thoroughly assessed the effectiveness and tolerability of ST as a primary treatment option in comparison to TT for the eradication of H. pylori. The analysis comprised a total of 15 randomized controlled trials, encompassing a sample size of 5,219 patients. The collective findings indicate that ST exhibits promise as it achieves higher rates of eradication. Additionally, it is worth noting that this approach has the potential to yield cost savings and enhance treatment compliance when compared to TT. To summarize, this systematic review and meta-analysis provide evidence that ST is a viable option for the initial treatment of H. pylori eradication. It shows potential benefits compared to the standard TT, especially when there is resistance to clarithromycin. In order to establish ST as the preferred first-line treatment, it is imperative that additional research be conducted to address the aforementioned limitations and thoroughly investigate its long-term efficacy and safety profiles. Nevertheless, it is required that additional research be conducted in order to adequately tackle the constraints of the current studies and solidify its position as a favored treatment alternative. It is also essential to consider ST as a viable approach to improve the rates of H. pylori eradication. This method should be thoroughly examined in clinical practice to gain a deeper understanding of its effectiveness.

## Introduction and background

Helicobacter pylori, a microaerophilic bacterium, is classified as a Gram-negative, spiral-shaped organism within the Helicobacteraceae family [1]. The H. pylori bacterium primarily localizes within the pyloric region of the stomach, thereby inducing a persistent gastric infection of a chronic nature. It is estimated that these bacteria have the potential to infect over 50% of the global population. The precise mode of transmission and infection of H. pylori remains somewhat elusive. However, it is widely believed that the fecal-oral and oral-oral routes, primarily through the ingestion of contaminated water or food, are highly implicated in its widespread occurrence. The incidence of H. pylori infection is observed to be on the rise in developing nations, exhibiting a substantial increase, as indicated by various studies [2-3]. The esteemed World Health Organization has officially classified H. pylori as a Class 1 carcinogen. The etiology in question is widely recognized as the primary factor contributing to the progression of gastritis, peptic ulcer, gastric ulcer, and gastric carcinoma, making it the most prevalent causative agent in these gastrointestinal conditions. According to estimations, there is a notable correlation between its consumption and elevated susceptibility to the development of peptic ulcer disease, with an approximate increase in risk ranging from 10% to 20% [4].

A multitude of therapeutic regimens has been subjected to meticulous evaluation via clinical trials to determine their efficacy in managing H. pylori infection. The integration of a synergistic combination of antimicrobial agents, specifically levofloxacin, metronidazole, amoxicillin, and proton pump inhibitors, has emerged as a well-established and essential aspect of modern therapeutic interventions [5]. The consensus within the medical community is that clarithromycin-based regimens represent the optimal therapeutic approach for triple treatment. The treatment of H. pylori infection has been associated with the emergence of antibiotic resistance. In terms of efficacy, it has been demonstrated in several trials that sequential treatment (ST) exhibits superior effectiveness compared to triple therapy (TT) [6-7].

The recommended treatment for H. pylori infection in Europe and the US continues to be a proton pump inhibitor (PPI) and clarithromycin with either amoxicillin or metronidazole. The elimination of H. pylori is an effective method of preventing stomach cancer and is the main method of gastric cancer prevention [8]. Despite this method's early successes, H. pylori eradication rates have fallen to under 75%. This is primarily due to the rise in metronidazole- and clarithromycin-resistant H. pylori strains, now a major global health concern [8].

Multiple studies have elucidated that the administration of sequential therapy over a duration of 10 days exhibits a superior preference when compared to the utilization of TT for either seven or 10 days. ST, a proposed alternative therapeutic strategy to conventional TT, has garnered attention in the medical community. The prescribed treatment regimen involves administering sequential therapy for a duration of five days. This entails the administration of a PPI twice daily, along with amoxicillin at a dose of one gram twice daily. This initial phase is then followed by a TT regimen, which includes the administration of a PPI twice daily, clarithromycin at a dose of 500 milligrams twice daily, and metronidazole twice daily, all for a period of five days [7-8].

Given the demonstrated higher elimination rate of TT, this evaluation aims to compare its safety and efficacy with ST as the initial management for H. pylori infection in individuals. This comprehensive analysis incorporates recent discoveries from rigorously conducted randomized trials in order to shed light on the potential benefits of treatment sequencing and its pivotal role in enhancing the eradication rates of H. pylori. Having a comprehensive understanding of the efficacy of sequential therapy in treating infections caused by H. pylori greatly informs medical decision-making and promotes the development of evidence-based approaches to patient management.

## Review

Methods

The present study employed the Preferred Reporting Items for Systemic Review and Meta-Analysis (PRISMA) 2020 guidelines to conduct a comprehensive systemic review [9].

Search Sources and Strategy

We conducted a comprehensive literature search using reputable databases, such as Medline, PubMed, Science Direct, and PubMed Central (PMC), to identify relevant scholarly articles. We conducted a comprehensive search across all databases utilizing diverse permutations of procalcitonin (PCT), C-reactive protein (CRP), sepsis, and postoperative period. Table 1 given below delineates the databases employed in the study, along with the corresponding quantified figures denoting the number of papers identified within each respective database.

**Table 1:** Search sources and strategy

Keywords/Search Strategy	Database Used	Numbers of Results
Helicobacter or Pylori AND Sequential AND Triple	Science Direct	1,649
Helicobacter or Pylori AND Sequential AND Triple	PubMed	376
‘’Helicobacter[all]’’ or Pylori AND ‘’Sequential[all]’’ AND ‘’Triple[all]’’ AND ((ffrft[Filter]) AND (randomizedcontrolledtrial[Filter]) AND (2015:2023[pdat]))	PubMed (Advanced Search)	20
The total number of research papers identified in this study is as follows.		2,045
The total count of articles after eliminating duplicates.		1,534

Inclusion Criteria

For this study, randomized controlled trials (RCTs) that had been solely written and published in the English language or had a readily available translated copy in English were selected. It was carefully decided to only select open-access and full-text papers, related to the field of medicine for this review. These research articles covered various age-related demographics. The patients, who had been selected and had received a positive diagnosis for H. pylori linked to other gastrointestinal conditions, were asked to undergo another confirmatory test. These confirmatory tests included the monoclonal stool antigen examination, the rapid urease examination (RUT), the histology or culture of any endoscopic biopsy specimen, or the urea breath examination (UBT). The main goal of these research papers was to do a comparative analysis between standard TT and ST in terms of their effectiveness in eliminating H. pylori.

Exclusion Criteria

For this study, all review articles, editorials, synthesis studies, observational studies, grey literature, and issued abstracts from our analysis.

Selection Process

Articles selected for inclusion in each database were exported to Endnote. We disposed of all copies of the file. Authors individually reviewed articles based on titles and abstracts. In the event of an eligibility dispute, all co-authors were consulted, and a decision was reached in consensus. The entire texts of the selected publications were reviewed again to ensure that only those that were relevant were considered. Only articles that met the exclusion and inclusion criteria were considered further.

Quality Assessment of the Studies

The quality of the articles that made the cut was evaluated with the help of appropriate quality assessment methods. All co-authors reviewed the quality of the work. Cochrane's assessment technique was used to assess RCTs. The systematic review only included studies that passed the quality assessment.

Cochrane Risk Bias Assessment Tool

**Figure 1:**
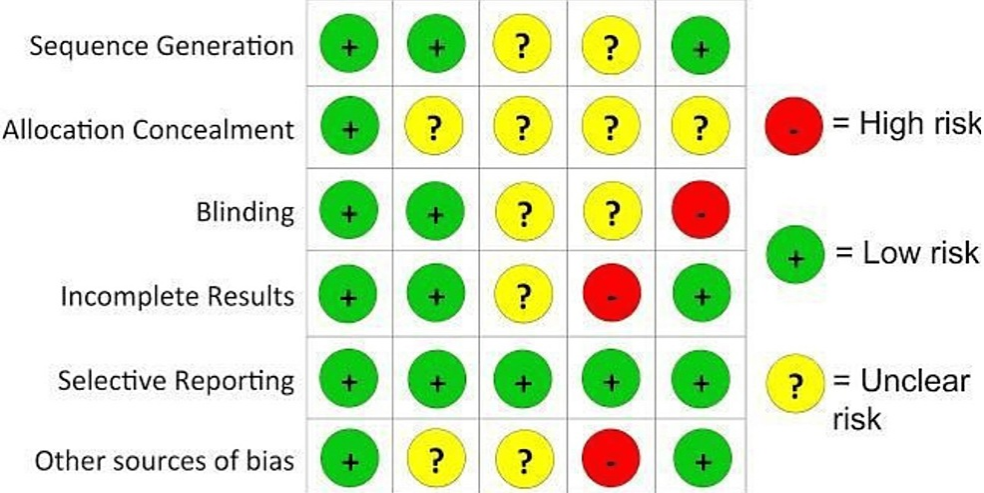
The detailed overall scores and quality for each study are provided in Table 1

The risk of the bias is shown below in the following Figure 1.

Data Collection Process

After the papers were selected and extracted for the systematic review, the primary outcomes and any other relevant data were analyzed. Each author contributed equally to the data extraction process and had a hand in finalizing the results.

Types of Interventions

ST

The prescribed treatment regimen, known as the 10-day ST, consists of a PPI and amoxicillin at a dosage of one g administered orally twice daily for the initial five days. Following this, it is recommended that the patient maintains their current regimen of taking the PPI twice daily in addition to clarithromycin at a dose of 500 mg twice per day, and a nitroimidazole at a dose of either 400 mg or 500 mg, both administered orally for the next five days.

We have incorporated clinical trials evaluating STs with a duration of 10 days. Exclusion criteria were applied to studies that exhibited any deviations in the mediation schedule pertaining to the duration of the SEQ treatment.

TT

The TT regimen encompassed a PPI, clarithromycin administered at a dosage of 500 mg twice daily, and amoxicillin administered at a dosage of 1 g twice daily. These medications were administered orally, and the treatment duration was a minimum of seven days.

Results

Study Identification and Selection

**Figure 2:**
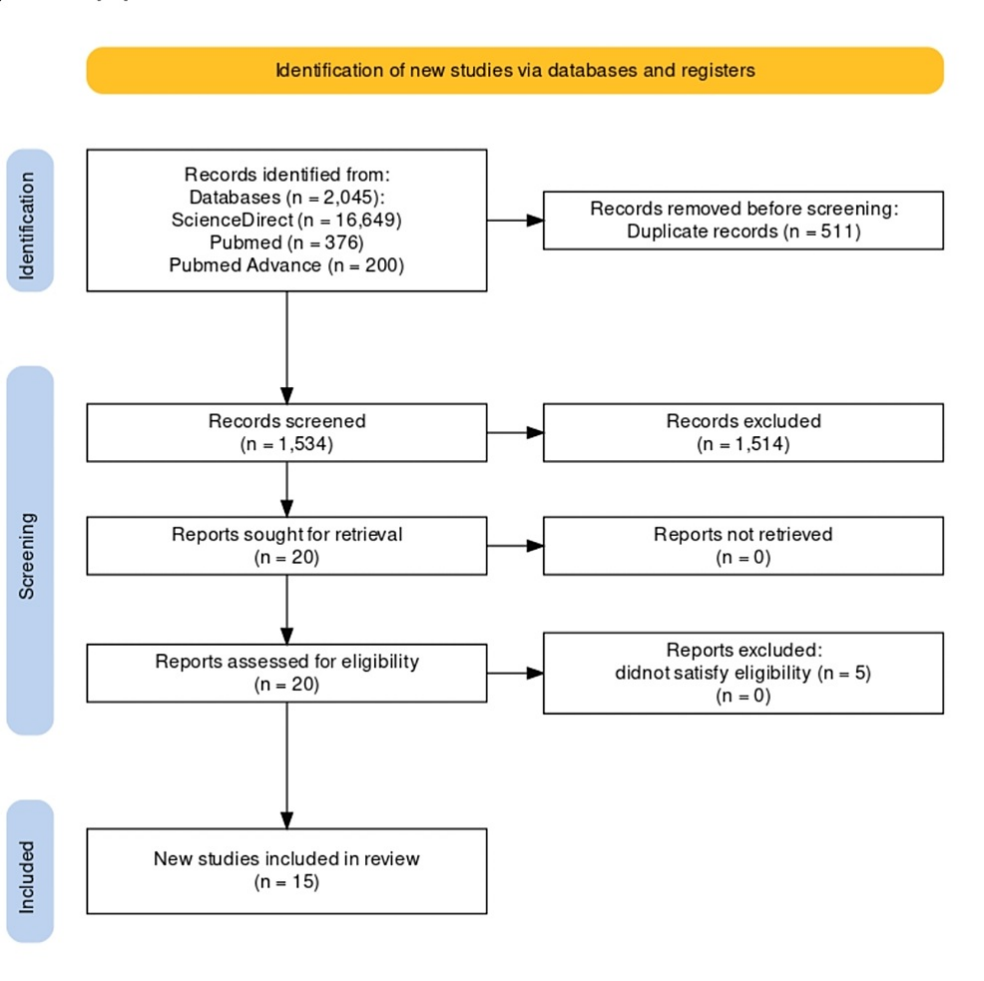
The PRISMA flowchart

Across all databases, we were able to narrow the pool of possible articles down to 2,045. After a thorough examination, 511 duplicate articles were eliminated. Twenty publications were selected for further review after an initial screening of titles and abstracts, followed by the retrieval of complete texts. After determining the eligibility and quality of the full-text papers that had been shortlisted, 15 were selected for the review process. The following Figure 2 of the PRISMA flowchart depicts the studies' selection procedure, whereas Table 2 shows the assessment of articles using the relevant Cochrane quality appraisal tools for eligibility [10].

**Table 2:** The articles were assessed for eligibility using the relevant Cochrane quality appraisal tools. *Key* *Risks of Bias* + = Low risk of bias - = High risk of bias ? = Unclear

Research	Random Sequence Generation	Allocation Concealment	Blinding of Participants and Personal	Blinding of Outcome Assessment	Incomplete Outcome Data	Selective Reporting	Other Bias
Su et al. 2022 [1]	-	-	-	-	+	+	+
AlRuthia et al. 2021 [2]	+	?	+	+	+	+	+
Kim et al. 2019 [3]	+	+	+	+	+	+	+
Zullo et al. 2019 [4]	+	+	-	-	+	+	+
Auesomwang et al. 2018 [5]	+	+	-	+	+	+	+
Ennkaa et al. 2018 [6]	+	+	+	+	+	+	+
Boal et al. 2017 [7]	+	+	+	-	+	+	+
Harmandar et al. 2017 [8]	+	-	?	?	+	+	+
Zhu et al. 2017 [9]	+	-	-	?	+	+	+
Iwańczak et al. 2016 [10]	+	+	?	-	+	+	+
Kim et al. 2016 [11]	+	+	-	-	+	+	+
Liou et al. 2016 [12]	+	+	+	-	+	+	+
Phiphatpatthamaamphan et al. 2016 [13]	+	-	+	?	+	+	+
Alsohaibani et al. 2015 [14]	+	-	+	-	+	+	+
Koroglu et al. 2022 [15]	+	-	?	?	+	+	+

Outcomes Measured

The primary outcomes extracted from the finalized research papers were the role of CRP and PCT levels, development of systemic inflammatory response syndrome (SIRS), sepsis, or death. The secondary outcomes that were evaluated included the duration of stay in the surgical intensive care unit (ICU), as well as the overall outcome of monitoring CRP and PCT levels. Several studies have investigated the impact of immunosuppressants on biomarkers and the progression of SIRS or sepsis. Furthermore, it was noted that there were relative changes in biomarkers when immunosuppressants were utilized.

Study Characteristics

We reviewed 15 RCT research papers with a total of 5,219 patients suffering from H. pylori. All studies involved H. pylori-positive patients, and the efficacy between TT and ST was compared. One study focused on associating the effectiveness of the 10- and 14-day sequential therapies, while another focused on the cost-effectiveness between the ST and TT.

Based on the findings of the systematic review, it can be inferred that ST exhibits potential as a primary treatment option for the eradication of H. pylori infection, in comparison to TT. The studies that have been reviewed consistently demonstrate that ST has the potential to attain higher rates of eradication, especially when dealing with strains of H. pylori that are resistant to clarithromycin. The observed impact of treatment durations in ST on patient outcomes is noteworthy, as longer durations exhibit a positive correlation with enhanced results. Furthermore, it is worth noting that ST has the potential to yield cost savings and comparable treatment compliance in comparison to TT. Nevertheless, it is crucial to acknowledge that the current body of research presents certain limitations, which encompass variations in treatment protocols and potential biases. Henceforth, the aforementioned findings underscore the imperative nature of conducting additional research in order to ascertain the enduring effectiveness and safety characteristics of ST as a favored therapeutic approach for the management of H. pylori infection. In general, the cumulative body of evidence substantiates the contemplation of ST as a promising alternative approach to augment the rates of H. pylori eradication. This warrants additional exploration and potential incorporation into the realm of clinical practice. The following Table 3 shows a summary and characteristics of all included studies.

**Table 3:** Summary and characteristics of all included studies.

Author and Year of Publication	Type of Study	Drug Studies	Purpose of Study	Number of Participants	Result/Conclusion
Su et al. 2022	RCT	Triple therapy and sequential therapy	The objective of this study is to examine the efficacy of 14-day sequential therapy and seven or 14-day triple therapy in eradicating infections in pediatric patients.	87	The eradication rate achieved by the 14-day sequential therapy was found to be significantly higher compared to the 7-day triple therapy (97.4% vs. 80%, p = 0.032) [1].
AlRuthia et al. 2021	RCT	Triple therapy and sequential therapy	This analysis aims to evaluate the financial viability and effectiveness of both treatment approaches.	170	The mean eradication rates observed for sequential therapy (SQT) and standard triple therapy (STT) were found to be 63.41% and 67.05%, respectively [2].
Kim et al. 2019	RCT	Sequential therapy and clarithromycin-containing triple therapy.	The aim of this study is to compare the effectiveness and safety of two different treatment regimens, namely 10-day CT and 10-day ST, with the standard 7-day clarithromycin-containing triple therapy (TT) as the initial treatment for Helicobacter pylori infection.	1141	The eradication rate achieved by the 10-day sequential therapy (10d-ST) protocol exhibited superiority over that of the 7-day triple therapy (7d-TT) protocol, as evidenced by the respective percentages of 76.3% versus 63.9% in the intention-to-treat (ITT) analysis, and 85.0% versus 71.4% in the per-protocol (PP) analysis [3].
Zullo et al. 2019	RCT	Sequential therapy	Examine the effectiveness of sequential therapy when administered over a duration of either 10 or 14 days.	291	The study's findings indicate that both the 10-day and 14-day sequential therapies have demonstrated a commendable success rate in eradicating Helicobacter pylori infection during initial treatment in real-world clinical settings [4].
Auesomwang et al. 2018	RCT	High-dose proton pump inhibitor-based triple therapy (HD-PPI-TT) and sequential therapy (ST)	In this analysis, we will be examining the effectiveness and tolerability of two different treatment options: HD-PPI-TT and ST.	120	The intention-to-treat eradication rates exhibited no significant difference between the standard therapy (ST) and high-dose proton pump inhibitor triple therapy (HD-PPI-TT) groups, with rates of 85% and 80% respectively (P = 0.47) [5].
Ennkaa et al. 2018	RCT	Sequential therapy and standard triple therapy.	The objective of this study is to compare the effectiveness and tolerability of sequential therapy (ST) as a primary treatment for Helicobacter pylori infection with that of standard triple therapy (TT).	206	The results of the intention-to-treat (ITT) analysis demonstrated that a treatment duration of 14 days using TT (Triple Therapy) yielded a slightly higher eradication rate compared to a 10-day treatment duration using ST (Standard Therapy) (54.8% vs. 50.7%) [6].
Boal et al. 2017	RCT	Sequential therapy and standard triple therapy.	The objective of this study is to compare the effectiveness of sequential therapy and triple therapy as the initial treatment for Helicobacter pylori.	60	The eradication rate achieved by sequential therapy was found to be 86.2%, while triple therapy demonstrated a slightly lower eradication rate of 77.4%. The p-value associated with this comparison was calculated to be 0.379, indicating that there was no statistically significant difference between the two treatment approaches [7].
Harmandar et al. 2017	RCT	standard triple, sequential, and quadruple therapies	In order to assess and contrast the eradication rates of Helicobacter pylori (H. pylori) using various therapeutic approaches, namely standard triple therapy, sequential therapy, and quadruple therapy, an investigation was conducted.	160	The successful eradication of H. pylori was observed in 28 out of 40 patients who underwent standard triple therapy, resulting in a success rate of 70%. Similarly, quadruple therapy led to H. pylori eradication in 33 out of 40 patients, achieving a success rate of 82.5% [8].
Zhu et al. 2017	RCT	The various treatment approaches under consideration include sequential therapy, triple therapy, sequential therapy in conjunction with Lactobacillus, and triple therapy in conjunction with Lactobacillus.	In order to assess the effectiveness of sequential therapy, triple therapy, sequential therapy in conjunction with Lactobacillus, and triple therapy in conjunction with Lactobacillus, a comparative analysis is required.	416	The most optimal therapeutic approach for the eradication of Helicobacter pylori infection in pediatric patients appears to be the utilization of sequential therapy in conjunction with the administration of Lactobacillus [9].
Iwańczak et al. 2016	RCT	sequential therapy and triple therapy	The objective of this study was to evaluate the efficacy and safety of both sequential and standard triple therapy regimens.	69	In pediatric patients afflicted with Helicobacter pylori strains that exhibited sensitivity to clarithromycin, the group receiving proton pump inhibitor (PPI) in combination with amoxicillin (AMO) and clarithromycin (CLA) achieved the most successful eradication rate (100%). Similarly, the group undergoing sequential therapy demonstrated a relatively high eradication rate (90.48%) [10].
Kim et al. 2016	RCT	sequential therapy and triple therapy	To compare the efficacy of ST with TT	601	The eradication rates for the intention-to-treat analysis were found to be 70.8% for the triple therapy (TT) group and 82.4% for the Sequential therapy (ST) group [11].
Liou et al. 2016	RCT	sequential therapy and triple therapy	To evaluate the effectiveness of sequential therapy over a period of ten days (S10) compared to triple therapy for a duration of 14 days (T14) as the initial treatment for Helicobacter pylori.	1300	The eradication rates of S10 and T14 were found to be 87.2% (567 out of 650, with a 95% confidence interval of 84.4% to 89.6%) and 85.7% (557 out of 650, with a 95% confidence interval of 82.8% to 88.2%) in the intention-to-treat (ITT) analysis, respectively [12].
Phiphatpatthamaamphan et al. 2016	RCT	Sequential therapy (SQT) and 14-day standard triple therapy (STT)	In order to assess the effectiveness of a 10-day sequential therapy (SQT) compared to a 14-day standard triple therapy (STT),	100	The eradication rate as determined by per-protocol analysis was found to be 97.9% (47 out of 48 cases) when utilizing the 10-day sequential quadruple therapy (SQT) regimen. On the other hand, the eradication rate was observed to be 87.8% (43 out of 49 cases) when employing the 14-day standard triple therapy (STT) regimen [13].
Alsohaibani et al. 2015	RCT	sequential therapy and triple therapy	In order to assess the effectiveness of sequential therapy as compared to standard triple therapy in the eradication of H. pylori infections, a comparative analysis is required.	195	The eradication rate achieved with sequential therapy was found to be 58 out of 93 cases, resulting in a percentage of 62.3%. On the other hand, standard triple therapy yielded an eradication rate of 69 out of 102 cases, corresponding to a percentage of 67.6% [14].
Koroglu et al. 2022	RCT	The various treatment options for Helicobacter pylori infection include bismuth-containing quadruple therapy (BQT), standard triple therapy (sTT), sequential therapy (ST), and hybrid therapy (HT).	In order to assess the efficacy of primary Helicobacter pylori eradication therapies, namely sequential triple therapy (sTT), bismuth quadruple therapy (BQT), standard triple therapy (ST), and hybrid therapy (HT), a comparative analysis is required.	303	The eradication rate of H. pylori in the intention-to-treat (ITT) analysis was observed to be 68.4% in the standard triple therapy (sTT) group, 79.5% in the bismuth quadruple therapy (BQT) group, 78.7% in the sequential therapy (ST) group, and 83.8% in the hybrid therapy (HT) group [15].

Discussion

H. pylori, commonly referred to as H. pylori, is a microorganism characterized by its Gram-negative nature, curved morphology, and possession of flagella. It predominantly colonizes the gastric region of the human body [11]. H. pylori infection has been established as a significant factor in developing various upper gastrointestinal diseases. These conditions encompass peptic ulceration, chronic gastritis, mucosa-associated lymphoid tissue (MALT) lymphoma, and gastric carcinoma. The eradication of H. pylori is not solely a crucial element in the therapeutic process of peptic ulcers. However, it is important to note that it also has a significant impact in reducing their reoccurrence, decreasing the chances of gastric carcinoma reoccurring after the removal of early gastric cancer, and aiding in the regression of MALT lymphoma [11]. The management of H. pylori infection encompasses a range of therapeutic approaches contingent upon several crucial features, including the patient's clinical manifestation, prevailing antibiotic resistance patterns, and unique individual considerations [11]. The principal objective of therapeutic intervention is to eliminate the H. pylori microorganism, thereby mitigating the likelihood of concomitant gastrointestinal pathologies, including gastritis, peptic ulcers, and gastric malignancy [12]. The main aim of this current systematic review was to perform a comparative analysis of the effectiveness and tolerability of ST as the initial treatment choice for H. pylori infection, as opposed to the standard TT, in terms of improving the eradication rate of H. pylori. The primary endpoints evaluated encompassed the rates of eradication achieved with ST and TT. In contrast, the secondary endpoints encompassed the treatment duration, cost-effectiveness, and safety profiles [12]. Moreover, the potential cost savings linked to ST, in contrast to TT, underscore its economic benefits. Furthermore, the comparable treatment adherence noted between ST and TT implies that ST is well-tolerated by patients.

TT

Since its introduction in 1996, TT with clarithromycin has become widely recognized as the preferred treatment for initial eradication, establishing itself as the gold standard. Several guidelines, such as the Maastricht IV, the Asia-Pacific Agreement Rules, and the Rule for H. pylori in South Korea, have shown a preference for a triple treatment strategy centered on the use of clarithromycin. Nevertheless, the effectiveness of the prevailing conventional TT regimen, which includes clarithromycin, has exhibited a distressing decline on a global scale, falling below the threshold of acceptability at less than 80%. It is postulated that the main cause of this deficiency is the development of bacterial resistance to clarithromycin. The decrease in the eradication rate of H. pylori is closely connected to the observed resistance rate toward clarithromycin [13].

ST

Based on the findings of Su et al. in 2022 [1], it has been noted that an ST regimen spanning 10 days has been previously employed. This therapeutic approach entails the administration of a PPI and amoxicillin for five days, followed by the subsequent administration of PPI, clarithromycin, and metronidazole for an additional five days. However, it is important to highlight that this treatment protocol is no longer recommended without susceptibility testing. This is primarily due to its diminished efficacy when confronted with strains of bacteria that have developed resistance [14].

Eradicating the Rate of ST and TT

This comprehensive review suggests that ST has demonstrated promising outcomes in eradicating H. pylori infection [15]. Several RCTs incorporated in this comprehensive analysis have documented superior eradication rates when employing ST in contrast to TT. A prospective observational study conducted by Hablass et al. encompassed a cohort of 303 individuals within the time frame spanning from July 2018 to June 2021. Sequential treatment was observed to have a 68.4% destruction rate, whereas triple treatment was seen to have a 74.3% destruction rate [16].

Similarly, a separate observational study conducted by Kim et al. [3] encompassed a cohort comprising a total of 1,141 patients. The protocol for concomitant therapy over 10 days demonstrated a significantly higher degree of eradication than the TT protocol over seven days. The efficacy in achieving eradication of H. pylori infection was found to be higher in the 10-day ST protocol compared to the seven-day TT protocol. The findings of this study indicate that ST could potentially serve as a superior therapeutic approach for managing H. pylori infection compared to the conventional TT regimen [17].

Based on the findings of Harmandar et al. in their 2017 study [8], it was observed that the utilization of 5 + 5 sequential therapies demonstrated a notably superior eradication rate of H. pylori when compared to the TT within the research group. A cohort comprising 160 individuals presenting with dyspeptic symptoms was recruited for the study. Among a cohort of 160 individuals diagnosed with H. pylori infection, it was observed that successful eradication was achieved in 131 patients, while 29 patients unfortunately experienced failure in their attempts to eliminate the H. pylori infection. The statistical analysis demonstrated that the ST consisting of a combination of two different treatment regimens administered consecutively, namely, 5 + 5, exhibited notably superior rates of H. pylori eradication when matched to the conventional TT [18].

Based on the findings of Razavizadeh et al. in 2020 [17], it was observed that sequential treatment regimens exhibited a higher rate of eradication in H. pylori infection compared to standard regimens. Henceforth, it was deemed a more suitable therapeutic regimen than conventional for initially treating H. pylori infection. Furthermore, this pharmacological treatment protocol demonstrated a decreased incidence of adverse reactions [6-19].

*Duration of Treatment of ST and *TT

The comparison of sequential and TT also took into account the duration of treatment [18]. The observational study conducted by Su et al. [1] demonstrated that a 14-day ST exhibited an eradication rate of 97%, in contrast to the 80% rate observed for a seven-day TT. A total of 38 patients underwent a 14-day ST regimen, while 24 patients were administered TT. A total of 25 participants were administered a seven-day triple treatment. The results of the study indicate that the 14-day ST exhibited superior efficacy compared to the seven-day TT in eradicating the specific infection under investigation. When comparing the eradication rate, it was observed that the 14-day ST exhibited superior performance in comparison to the 14-day TT. According to the study conducted, it has been found that a 14-day ST regimen is more effective compared to TT in treating pediatric patients who are newly diagnosed with H. pylori infection [19]. ST exhibits an eradication rate of 90%, rendering it highly effective in regions where clarithromycin resistance is prevalent. The 14-day ST exhibited a superior eradication rate in comparison to the 14-day TT, albeit lacking statistical significance. The aforementioned findings indicate that the use of prolonged ST may have a positive impact on the eradication rates of H. pylori. This underscores the significance of the duration of treatment, as it plays a crucial role in achieving successful outcomes [19].

In a similar vein, a supplementary observational study conducted by Liou et al. in 2018 [12] examined a cohort consisting of 1,300 adult individuals who were affected by H. pylori infection and had not received any prior treatment. The subjects were assigned randomly in a one-to-one ratio to receive ST for a period of 10 days (referred to as S10). ST involved the administration of lansoprazole and amoxicillin for the initial five days, followed by lansoprazole, clarithromycin, and metronidazole for the subsequent five days. Alternatively, they could undergo TT for a duration of 14 days (designated as T14). TT entailed the administration of lansoprazole, amoxicillin, and clarithromycin for the entire 14-day duration. The administration of medications took place on a bi-daily frequency. Ever since the publication of Liou et al.'s groundbreaking research in 2018, it has been widely acknowledged within the scientific community that receiving ST for a period of 10 days does not demonstrate any superiority over TT for a duration of 14 days in terms of clarithromycin inhibition, as observed in the specific levels they investigated [19].

According to research conducted by Ennkaa et al. in 2018 [6], it was noted that the administration of a 10-day ST resulted in a higher rate of eradication of H. pylori compared to a 10-day conventional TT [20]. However, it is worth noting that both ST and TT have proven to be ineffective in achieving desirable eradication rates in specific regions where there are high levels of resistance to clarithromycin and metronidazole. Hence, it is evident that the 14-day ST regimen demonstrated a significantly higher eradication rate when compared to both the seven-day TT and the 10-day ST [19].

Cost-Effectiveness of ST and TT

In relation to cost-effectiveness, the investigation by AlRuthia et al. [2] delved into the economic implications linked to ST and TT. The results of their investigation indicate that ST demonstrates a higher likelihood of cost savings and improved effectiveness when compared to TT. The study revealed that the mean costs related to ST were comparatively lower when compared to TT. Furthermore, it is noteworthy that the efficacy rates in eradicating the specific ailment via ST, recorded at 63.41%, exhibited a resemblance to the outcomes attained through TT, which accounted for 67.05%. The aforementioned observations suggest that ST can produce more favorable results in effectiveness and cost-efficiency when utilized as the primary treatment strategy for controlling H. pylori infection [19-20].

Safety Profiles of ST and TT

According to Auesomwang et al.'s [5] observation, there were two therapy groups for the 120 individuals with H. pylori-related functional dyspepsia. Rates of eradication, antibiotic resistance, dyspeptic symptoms, treatment compliance, and side effects were compared. In relation to safety profiles, the study conducted by Auesomwang et al. [5] in 2018 revealed that ST exhibited a superior eradication rate compared to high-dose proton pump inhibitor-based TT (HD-PPI-TT) when analyzed on a per protocol basis. This observation implies that ST might exhibit enhanced efficacy, especially in instances involving H. pylori strains that have developed resistance to clarithromycin. The treatment adherence was comparable between the ST and TT regimens, albeit with a higher prevalence of nausea and dizziness observed in the ST cohort. Additional research is required to further investigate the safety profiles and adverse events linked to ST to evaluate its tolerability more accurately [15].

Limitations

It is imperative to acknowledge that this systematic review possesses certain limitations. First and foremost, it is important to note that the studies included in this analysis exhibited variability in sample sizes, treatment durations, and follow-up periods. This variability may introduce heterogeneity, which should be considered when interpreting the results. Furthermore, it is imperative to acknowledge that the aforementioned studies were carried out in diverse geographical regions. This factor could potentially influence the observed rates of antibiotic resistance and the outcomes of treatment interventions. In addition, it is important to note that certain studies were identified as having a heightened risk of bias or an indeterminate risk of bias during the quality appraisal process. This particular observation can potentially impact the overall validity of the research findings. Ultimately, the scope of the investigation was constrained to publications written exclusively in the English language, thereby potentially introducing a linguistic bias into the study.

## Conclusions

In summary, the outcomes of this comprehensive analysis offer significant insights into the potential efficacy of ST as an initial therapeutic approach for eliminating H. pylori infection. The literature reviews indicate that ST may yield superior eradication rates, particularly in instances with a high prevalence of clarithromycin resistance. The observed correlation between the extended duration of ST and enhanced treatment outcomes suggests that the length of treatment plays a crucial role in eradicating H. pylori. Generally, ST exhibits promising potential as an alternative approach to augment the rates of H. pylori eradication. 

Upon careful examination of the studies included in this systematic review, it becomes apparent that ST exhibits considerable potential as a primary treatment option for the eradication of H. pylori infection, in comparison to TT. The studies that have been reviewed collectively indicate that ST has the potential to attain higher rates of eradication, particularly in instances where there are clarithromycin-resistant strains of H. pylori present. This clinical advantage holds considerable importance in the field. In order to establish ST as a preferred option, it is imperative to conduct additional research to thoroughly examine its long-term efficacy and safety profiles. As a medical student pursuing advanced studies, this analysis emphasizes the significance of employing evidence-based decision-making within clinical settings. It also brings attention to the necessity of conducting more extensive research to enhance the effectiveness of H. pylori eradication strategies.
